# Using city gates as a means of estimating ancient traffic flows

**DOI:** 10.1371/journal.pone.0229580

**Published:** 2020-02-27

**Authors:** J. W. Hanson

**Affiliations:** Department of Classics, University of Reading, Reading, Berkshire, United Kingdom; National Taiwan University, TAIWAN

## Abstract

Despite the recent flurry of interest in various aspects of ancient urbanism, we still know little about how much traffic flowed in and out of ancient cities, in part because of problems with using commodities as proxies for trade. This article investigates another approach, which is to estimate these flows from the built environment, concentrating on transport infrastructure such as city gates. To do this, I begin by discussing a new model for how we would expect this kind of infrastructure to expand with population, before investigating the relationship between the populations of sites and the total numbers and widths of city gates, focusing on the Greek and Roman world. The results suggest that there is indeed a systematic relationship between the estimated populations of cities and transport infrastructure, which is entirely consistent with broader theoretical and empirical expectations. This gives us a new way of exploring the connectivity and integration of ancient cities, contributing to a growing body of general theory about how settlements operate across space and time.

## Introduction

Although there have been considerable advances in our ability to investigate various aspects of human life through the collection of large amounts of data, it can still be a significant challenge to estimate socio-economic flows because of the wide range of channels through which these exchanges can occur [1]. This is in part because of the difficulties associated with identifying and distinguishing between the spatial and socio-economic boundaries of cities, especially in the presence of the high levels of commuting brought about by advances in transportation systems, and in part because of the difficulties of capturing appropriate data at appropriate scales of analysis [1]. As a result, while most recent models assume that there is some relationship between the sizes of cities and the rates and volumes of traffic flows, it is still not clear whether there is a systematic relationship between them and, if so, what it is [1,2]. This article explores whether it is possible to investigate these issues using historical and archaeological data. Although ancient data might not be as abundant as modern sources, they can make an important contribution to the discussion, given that we would not only expect commodities and information to have been exchanged physically owing to lower levels of technological development including transportation systems, but also for these exchanges to have required physical infrastructure that can be measured quite readily from material remains.

Despite the recent flurry of interest in various aspects of ancient urbanism, we still know little about how much traffic flowed in and out of ancient cities, in part because of issues with assessing the scale of daily commuting and seasonal migration, as well as problems with using commodities as proxies for trade. As Killgrove and Montgomery have pointed out, although it is clear from both literary and epigraphical evidence that the movement of individuals was a regular occurrence in ancient life, it is more difficult to quantify its exact scale [3]. An obvious new line of evidence is bioarchaeological material, as shown by recent work on cemeteries in Rome from the Imperial period, which suggests that immigrants did indeed constitute a significant proportion of the inhabitants of the capital [3]. Unfortunately, it is more difficult to extend this work to other sites, given the scale of the available evidence and the cost of the procedures involved. In addition, this evidence cannot be used to give us a sense of daily or even seasonal flows in and out of cities, since it only reflects the people who came to them, stayed for whatever reason, and ultimately died.

Another approach has been to use small finds, such as ceramics, as a reflection of the overall scale of trade [4, 5,6]. It is often impossible to collect quantities at the site level, however, since very few sites have been extensively cleared and excavated, leading to inevitable concerns about the representativeness of the material we do have. There are also challenges in extrapolating from the observed assemblages to the total amount that was originally exchanged. This stems from uncertainties about not only what has entered the material record in the first instance (including the extent to which vessels were reused and recycled and the nature of the move to more perishable containers, such as barrels and skins), but also about how this material has been collected, arising from inconsistencies in the recording of different classes of material. We have much better records for fine wares than coarse wares, simply because of their relative usefulness for dating and the sheer quantity of material that remains. This means that it is difficult to compare the ratios of different classes of material in sites, such as the relative numbers of imported to local wares or the relative numbers of different kinds of vessels, in more than a handful of cases. As van Oyen has pointed out, for example, it is clear that the ‘consumption of [terra] sigillata in cities in Britain was both quantitatively and qualitatively different from patterns at non-urban sites’, yet there is not enough evidence for the volumes of different ceramics to allow us to identify a systematic relationship between the sizes of sites and the relative rates of consumption of these commodities [7: 291].

This means, in general, that the extent to which individual communities were dependent on daily commuting or seasonal migration or on commodities obtained from beyond their immediate hinterlands is far from clear. This gap in our understanding is obviously important to address, since a clearer understanding of these issues should have important implications not only for our view of the economic roles of ancient cities and the extent to which they were able to specialize or diversify at both local and regional levels, but also for wider debate about the nature of daily commuting and seasonal migration in pre-industrial settings and the extent to which the expansion or contraction of settlements in non-modern contexts mainly occurred through preferential attachment or random processes [8].

Here, I explore another approach, which is to use the built environment itself to estimate traffic flows. This is only possible because of recent research, which is based on a conception of settlements as social networks embedded in the built environment, allowing us to design a series of models for the relationship between the estimated populations of sites and their infrastructure and socio-economic conditions (and how these attributes change as the number of people involved increases) [9, 10, 11, 12, 13, 14, 15,16]. One of the most important implications of these theories is that we would not only expect there to be a relationship between the populations of sites and various aspects of their built environments and socio-economic conditions, but also for these two sides of urban life to be relatable to one another, allowing transport networks and traffic flows to be considered in the same scheme. These theories have been applied to various aspects of sites in both urban and rural contexts and in both contemporary and ancient settings, but there has so far been little attempt to look at outward-facing infrastructure, such as that designed to accommodate the flow of traffic in and out of settlements.

An important exception is Altaweel and Palmisano’s discussion of sites in the Khabur triangle in northern Mesopotamia in the Late Chalcolithic and Bronze Age (ca. 4200–1200 BCE), which concentrated on the mound areas and mean hollow way widths (i.e. the remains of roadways) of a selection of sites [17]. While they did find a relationship between these features, its exact nature varied substantially for different groups of sites. Although the authors are cautious about the wider applicability of these results, they are nonetheless promising since they can be used as a reflection of the scale to which larger settlements might have acted as greater social attractors or had more intensive economic activity relative to smaller sites, giving us a first glimpse of how settlements and agricultural landscapes could have interacted in formal terms.

This therefore raises the question of whether we can extend Altaweel and Palmisano’s work by using other forms of transportation infrastructure as a proxy for traffic flows and, in particular, whether we can identify any relationship between the sizes of ancient cities and the amount of traffic that passed through them. Greek and Roman cities are an ideal candidate for this, since they are not only uncommonly well documented, but were also often surrounded by physical walls, meaning that all traffic would have had to pass through city gates, which can be measured directly from maps and plans.

## Aims and objectives

This article investigates whether the built environment can be used to estimate traffic flows. It begins by reviewing current understanding about the sizes of cities and traffic flows and discussing a new model for how we would expect transport infrastructure, such as city gates, to expand with population. It then tests the hypothesis that the former will increase more slowly than the latter by exploring the relationship between the estimated populations of sites and the total numbers and widths of city gates, before examining their relationship with other network measures, such as degree centrality, betweenness centrality, and Eigenvector centrality, using ORBIS: The Stanford Geospatial Network Model of the Roman World (henceforth the ORBIS model) [18]. It shows that there is a systematic relationship between the estimated populations of cities and transport infrastructure, which is entirely consistent with expectations about how traffic flows expand with population. This gives us a new way of grappling with how connected and integrated ancient cities were, which might allow us to contribute to a growing body of general theory about how settlements operate across space and time. The unit of analysis is cities, focusing on the Roman world in the Imperial period.

## Theoretical background

A major area of interest in urban studies is how flows in networks scale with size [19]. Although it has been obvious for some time that there is a link between the sizes of cities and their wider influence, one of the most important implications of recent work is the observation that cities can be characterized as venues for dense local interactions, imbricated in complex long-distance movements of people, goods, and information [19]. This is important because it means that we would expect the process of agglomeration to lead to systems of linked, but specialized, cities, operating at various scales of resolution from national to global [19, 20,21]. In this situation, we would expect there to be an increase in both the extent of face-to-face social interactions and the scale of long-distance travel [19].

There is also evidence for various kinds of scaling in modern transportation systems, based on the number of locations that can be accessed within a certain travel time budget [1]. Commuting times tend to be about an hour (half an hour in each direction), regardless of the sizes of the cities in question or the modes of transport available, and tend to be log-normally distributed, suggesting that improvements in technology have been used to extend the distances travelled, rather than to reduce the time spent travelling (known as Marchetti’s constant) [1,22]. Meanwhile, most traffic within cities concentrates on a small fraction of streets, so the distribution of traffic flows over them can be well characterized by a power law [1,23]. Interestingly, this also seems to extend to traffic beyond cities, since, while every vehicle has to use the roads coming in and out of cities, we would then expect them to fan out across the country by branching off onto smaller and smaller roads, creating a fractal-like pattern, as demonstrated by recent empirical work on the present day interstate road network of the US [1]. Finally, there is also evidence that the number of visitors to a given location scales inversely as the square of both the distance travelled and the frequency of visitation [1]. Although these observations are based on contemporary experience, they are nonetheless important for historians and archaeologists, since they suggest that we can observe traffic flows in settlements at any level, so long as this is done consistently. It also has useful implications for the model discussed below, since it suggests that, while we would expect roads to have radiated from settlements in all directions, we would also expect one or two roads to have carried most of the traffic.

The most well-known way of representing these ideas is the gravity model [24]. Although the model was originally designed to estimate bilateral trade flows based on the economic sizes, i.e. the GDP, and the distances between two countries or regions, it has also been found to be a useful tool for studying a wide range of other phenomena, including migration and traffic flows. This has led to a series of attempts to use the model to study various ancient phenomena, including, most recently, the spread of Christianity between different communities in the Roman Empire [25, 26, 27,28].

The model is based on the idea that the estimated degree of interaction between two entities, such as two cities, increases with their sizes and decreases with the distance between them: $g_{nm}=\frac{N_{n}^{\alpha }N_{m}^{\beta }}{d^{\gamma }}$, where *N*_*n*_ and *N*_*m*_ are the sizes of the interacting entities, *α* and *β* are measures of the propensity of individuals to move or exchange commodities, *d* is a measure of the distance between them (but this can also be substituted with an estimate of the cost of travelling between them on different networks, such as on sea, river, and road routes), and *γ* is a measure of friction [28]. The model can then be adapted to estimate various other aspects of sites, simply by replacing the second term in the numerator with another variable.

Although the model has been successively used to characterize both ancient and modern cities, there is still considerable uncertainty about exact values of the exponents, mostly surrounding the observed variability in the value of the *γ* parameter, which seems to range from anywhere between 0.5 to 3 (perhaps depending on the nature of the system investigated) [28]. As a result, despite the fact that the gravity model has been fundamental to our understanding of the relationship between the sizes of sites and the magnitude of traffic flows for nearly 50 years, it is still not possible to use it to estimate ancient traffic flows *per se*, even if we do know the size of cities and the distances between them.

In this article, I offer an alternative approach, which is to build a model for traffic flows based on the design of cities themselves. This is grounded in recent theoretical and empirical work on complex systems, known as settlement scaling theory, which consists of a set of models that explain how key quantitative attributes of settlements are predicted by their populations [9, 10]. The models are based on the increasing intensity and frequency of social interactions that come from the concentration of people in space and time, after taking account of the costs and benefits of interaction. These models have the generic form: $Y_{t}=Y_{0}N_{t}^{\beta }$, where *Y* is the measure under consideration, *N* is the population, *Y*_0_ is a prefactor (or constant), *β* is an exponent, and *t* is a subscript denoting a particular time, with infrastructure tending to grow slower (i.e. *β* < 1) and socio-economic rates tending to grow faster (i.e. *β* > 1) [29]. Road surfaces in contemporary cities, for example, tend to increase with *N*^(1−*δ*)^, while the total number of telephone calls increases with *N*^(1+*δ*)^ [9,30]. It is worth pointing out at the outset, however, that these models are only intended to capture the average relationships across sites and do not necessarily allow us to make predictions about specific sites (which are obviously conditioned by various local factors).

An important component of these models is that individuals in larger cities will not only live at higher densities, but also have more social contacts [9, 10]. As we have shown elsewhere, this can be used to suggest a model for how we would expect the mixing space of cities to change as they increase in size by considering the amount of space required for a community of a certain size to interact in pursuit of their daily needs and how this space will expand as the numbers of individuals involved increases [15]. This can then be used to estimate how we would expect the infrastructural areas of cities to expand with population, helping to explain the quantitative patterns in both public spaces (*fora* and *agorae*) and street networks observed in cities in the Roman world in the Imperial period [15].

This model suggests that the total area given over to transport infrastructure should expand with *N*^(1−*δ*)^, with the widths of streets increasing slightly slower than their lengths [15]. Given that both theoretical and empirical observations demonstrate that the value of δ for Greek and Roman cities is about 1/3, we would then expect the exponent to be 2/3, with widths taking about 1/6 and lengths around 1/2 [13, 15]. We would therefore expect city gates to have increased at about the same rate, given that they were an important part of the transport infrastructure. However, since we are only interested in their linear dimension (i.e. the total width of city gates), not their areal dimension (i.e. the total area), we then take the square root, giving us *N*^(1−*δ*)/2^, suggesting an exponent of about 1/3.

At this point, we should be able, at least in theory, to estimate the actual flux (i.e. the rate of flow), using the following formula: *q* = *ku*, where *q* is the flux, *k* is the density (i.e. a combination of how many pedestrians or vehicles could travel abreast and how closely they followed one another), and *u* is their speed. Unfortunately, it is not possible to do this, given our ignorance about both the speed and density of traffic. Although we can gain a sense of the average widths required for pedestrians or vehicles, such as from the remains of the wagon from Stabiae and ruts from sites, we obviously have no way of knowing how frequently they passed through city gates. Having said this, we can assume that both speed and density are independent of the sizes of cities, meaning that the only way to increase flux across cities would have been to increase the width of city gates.

Finally, since we would expect the city gates to be an expression of the average amount of traffic that could have come and gone over the course of one day, including both slack times and periods of intense activity (with possible tailbacks), we would also expect the total numbers and widths of city gates to be a reflection of the average rate of traffic flow *per diem*, rather than the total flow over the same unit time. Having said this, given that our evidence for each site pertains to the entire settlement, it is also reasonable to assume that the total numbers and widths of city gates would also have been set by how much the average individual used them, making them effectively a *per capita* measure. If this is correct, then we should be able nonetheless to calculate the total flow by multiplying the average rate by the size of the population, in keeping with wider settlement scaling theory. As a result, if the total width of city gates increases with *N*^(1−*δ*)^, i.e. *N*^1/3^, then the total flow (in and out per unit time) will increase with *N*^2(1−*δ*)^, i.e. *N*^4/3^. Although this might appear to introduce collinearity effects, it should be emphasized that it will not affect the regression results presented below, which are based solely on the total numbers and widths of city gates (and therefore only involve population on one side). The only question is therefore whether we can make the interpretative leap from the average to the total. This is discussed below.

## Data and methods

### Numbers and widths of city gates

Although walls did have a defensive purpose, they also had an important role in helping to control and regulate traffic by channelling the movement of people, goods, and ideas into and out of cities. We would expect ancient architects to have struck a balance between defensive and logistical needs, creating enough city gates to serve material needs, without sacrificing defensive concerns. Although some scholars, such as van Tilburg, have been skeptical of their overall functionality for facilitating traffic flow, there are now several grounds for regarding these structures as an effective part of the transportation infrastructure [31]. As Poehler has argued in a ground-breaking study of the traffic systems of Pompeii and a selection of other sites, there is evidence for the control and regulation of traffic, as shown by the preference for right-sided driving and the existence of one-way streets, as well as for the management of congestion through simple mechanisms such as traffic segregation [32]. This reflects an overall concern for making cities inhabitable and functional environments. In addition, it is clear that the general trend was for larger city gates with more passages connected to wider streets, progressing from narrow one lane city gates to massive structures with as many as four passages, echoing other evidence for the sizes of cities during the same period [31, 32]. These developments are illustrated by Pompeii, beginning with the Porta di Stabia (which was only wide enough for one lane of traffic to move in one direction and in its current form dates mostly to the third century BC) and culminating in the Porta Ercolano (which had multiple openings for pedestrian and vehicular traffic and roughly reflects conditions around the time of the eruption of Vesuvius in AD 79) [32]. Moreover, city gates could be added or removed (e.g. Rome, the Porta Clausa), and rebuilt (Pompeii, the Porta di Stabia) and there is evidence that the reconstruction of city gates went hand in hand with the reconstruction of streets. As Poehler notes, for example, at Pompeii ‘the (re)construction of gates was accompanied by the provision of a new, durable, and aesthetically pleasing surface to support the traffic they were envisioned to carry’ [32: 62].

One of the main advantages of these features of walls is that they are very common: about 64% of the cities that have been documented in the Roman world in the Imperial period had walls at some point in their histories [12]. Along with street networks, walls also tend to be one of the most durable features of ancient cities in any context and many stretches still survive, despite more recent occupation. This is in part because of their deterministic effect on street networks and design of the urban layout and in part because of their ongoing usefulness in defensive terms: the Aurelian Wall, for example, was still part of Rome’s defences as late as the 19^th^ century. Although there is an important inflection point with the onset of the Industrial Revolution, when walls and gates were increasingly dismantled or demolished to improve traffic circulation alongside increasing demographic growth, industrialization, increasing military capabilities, and changes in transport technology, both walls and gates are often preserved in the modern layout. This means that we should at least be able to count the numbers of city gates, even if we are not able to measure the dimensions of every single one. We can therefore be confident that this material will be more tractable than other forms of evidence, such as small finds, and that we will encounter fewer difficulties relating to the overall representativeness of the available material.

To estimate the amount of traffic that could have been accommodated by these city gates, I measured both the total numbers and widths of city gates from an existing collection of maps and plans, standard architectural manuals, and individual articles and monographs on selected sites, concentrating on the list of sites given in an earlier catalogue of cities and towns [12]. These resources include reconstructions of the locations of city gates and the courses of ancient walls, which are usually based on a combination of the layout of the modern settlement, published excavation reports, and unpublished archival material.

Although the earliest city gates only had one portal, later ones usually have separate portals for pedestrians and vehicular traffic, which were distinguished by both their relative numbers and widths and their different surfaces [31, 32,33]. The simplest city gates took the form of a passage that could be closed with either one or two sets of city gates at each end, while more complex versions included a central courtyard that occasionally also included a portcullis [31]. As Poehler has pointed out, these developments are important, since they not only allowed traffic to be divided by type, i.e. into pedestrian and vehicular, but also for it to be divided by direction, so that traffic entering and exiting would not have to meet [32]. At this point, it is also worth commenting on the common assumption that daytime travel was banned for certain vehicles [31]. As Poehler has argued, this injunction can best be seen as a blunt instrument that divided traffic into two types, vehicular and pedestrian, effectively restricting one for the improvement of all [32]. I therefore included both kinds of portals, since we are interested in all flows in and out, regardless of the mode of transport, direction of travel, or time of day. I have measured city gates at their narrowest points, since we are concerned with the amount of traffic that could have come and gone at any given moment (i.e. in the choke-points that determined the maximum amount of traffic that could be accommodated). I have not included posterns, however, on the assumption that they were mainly used for official purposes or in exceptional circumstances, such as in the event of a siege. In any case, the effect on the final relationships is negligible.

Since it is not always possible to measure all the city gates in each site, I attempted to measure both the numbers and widths of city gates and then multiplied their average width by the total numbers of city gates. Although this will make no difference to the sites with complete information, it should be the best way of extrapolating from known to unknown in the sites with less evidence. It is worth pointing out, however, that one of the limitations of this approach is the small numbers of estimates that we have for some sites, which raises the question of whether the averages that have been used are reliable. Although it would be convenient if most of the city gates in each settlement had a reasonably uniform design, given that they were normally part of the same defensive scheme, this is unlikely given the expectation that traffic is normally not distributed equally across roads and therefore across city gates. This should not be an issue for this article, however, since we are interested in the average across all settlements, not individual ones. This assumption also seems to be borne out by the fact that both the total numbers and average widths of city gates, as well as the maximum widths of city gates, all obey similar relationships.

Finally, it should be pointed out that, for the purposes of this article, I have mostly focused on inland sites, for the obvious reason that we would expect a great deal of the traffic to and from coastal sites to have gone through harbours, especially if it was possible to get from the harbour to the settlement without going through city gates. This is something that might be expanded on in future work, assuming that it is possible to derive detailed estimates of the amount of traffic that could be accommodated by harbours.

The next issue concerns the estimated populations of sites. Until recently, scholars have been almost completely ignorant about the sizes of ancient cities, given the lack of available census data and inherent issues with the small assortment of figures that have come down to us in textual sources (which often only refer to certain privileged groups or include the residents of both urban and rural areas) [12, 13,34]. In the last decade or so, there has been a significant breakthrough in our ability to estimate the populations of sites through advances in our evidence for their sizes and densities, which allow us to estimate their populations by simply multiplying the former by the latter [12, 13, 34]. I have therefore used the same method to estimate the populations of sites, following previous papers [12, 13, 14, 15, 16]. These estimates are based on a combination of the inhabited areas (i.e. the maximum extent of the contiguous remains of each site) and densities of sites, using the average relationship between the inhabited areas and housing densities of sites derived from a sample of over 50 sites throughout the Greek and Roman world where it is possible to count the numbers of residential units per unit area and estimate the sizes of their households to create a bespoke estimate for each one (Table 1). One of the main advantages of this approach is that it supplies evidence for the estimated populations of sites, which is independent of their transport infrastructure, allowing us to examine the relationship between these variables for the first time. This, in turn, allows us to examine how the sizes of their population relates to traffic flow.

**Table 1:** The relationship between the estimated populations and inhabited areas of a selection of sites in the Roman Empire (after Hanson and Ortman 2017: Table 5).

Dependent variable	Number of cases	Exponent (95% CI)	Pre-factor (95% CI)	R^2^	Significance (P value)
Inhabited area (ha)	53	0.654 (0.587–0.721)	0.146 (0.078–0.274)	0.877	<0.0001

It does not matter if I have used the same walls to estimate the numbers and widths of city gates and the sizes of cities, since I have always attempted to measure the actual built-up area if feasible. However, I have generally avoided using third century walls, since they were often built as part of a general response to the political insecurity and economic downturn that occurred in the late third century, so these features often include a smaller area than originally inhabited. Lastly, many of the estimates date to different periods. This should not affect the overall results, since the data for both the sizes of sites and the numbers and widths of their city gates is most likely to reflect the period of peak occupation, with the result that, although these data have different effective dates, they nonetheless reflect the same properties in each case. In addition, there is now substantial evidence that even sites from different periods will follow a single scaling relationship, provided they are part of the same system.

**Fig 1:**
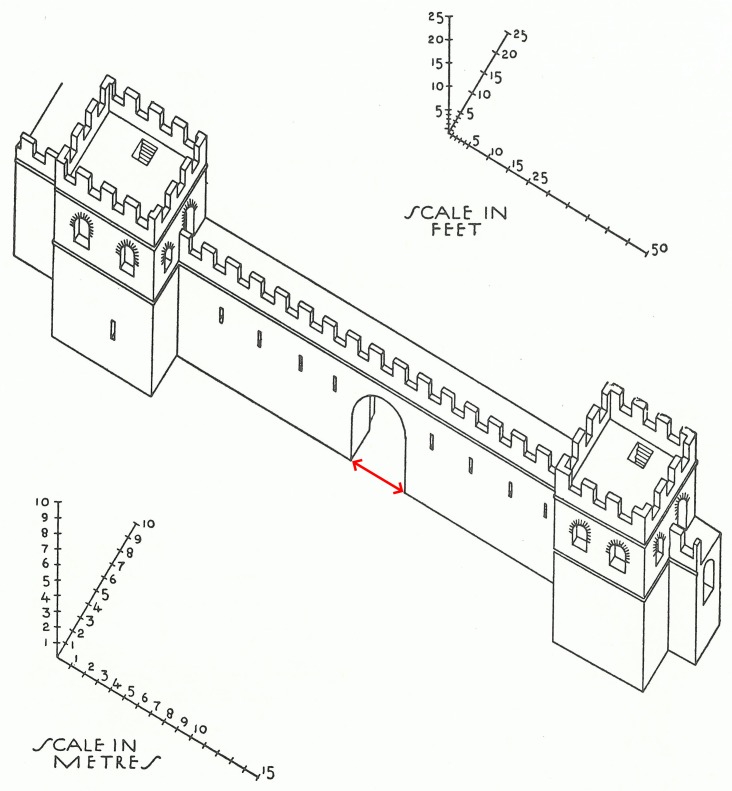
An illustrative example of the features used in this study, in this case one of the city gates, the Porta Asinaria, in the Aurelian Wall, Rome (after Richmond 1930: Fig 27). The red arrow indicates the section that has been measured, which is the width of the city gate.

**Fig 2:**
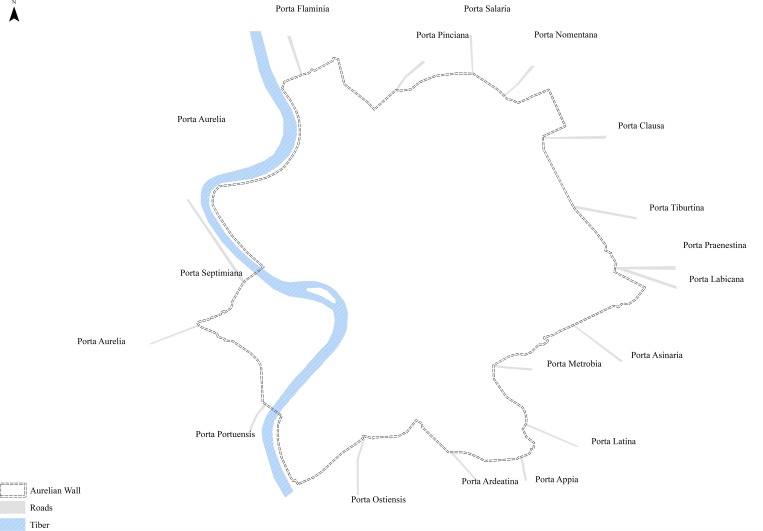
City gates in the Aurelian Wall, Rome.

Although the capital was not defended during its maximum extent in the second century AD, a circuit of walls, known as the Aurelian Wall, was thrown up in short order between AD 271 and 275 in response to the barbarian invasion of AD 270 [35, 36,37]. These walls did not enclose the entire inhabited area and often took the path of least resistance (sometimes cutting through vacant lots and sometimes incorporating existing buildings), but did encompass the most important neighbourhoods including the original nucleus of the settlement, the Campus Martius, and the left bank of the Tiber, running for 19 km and enclosing 1,373 hectares. As Richmond has shown, these walls were remodeled several times in the third, fourth, and fifth centuries AD, but in their final form they had 18 main city gates and 5 postern city gates, which were themselves adapted several times, taking slightly different forms in each instance (Figs 1 and 2) [35, 36, 37]. We can measure the widths of exactly half of these city gates, including the Porta Flaminia (one portal), Pinciana (one portal), Nomentana (one portal), Tiburtina (one portal), Praenestina (two portals), Labicana (two portals), Asinaria (one portal), Appia (two portals), and Ostiensis (two portals), suggesting an average width of just under six metres. It should be pointed out, however, that we do not have any evidence for the left bank. As noted above, although these walls did not enclose the entire inhabited area at the time of their construction, I have used this area as the basis for the estimate of the population, given the evidence that whole quarters were left outside the walls and progressively abandoned [36, 37]. Since these walls include 1,373 hectares, the estimated population is 651,000 people [12, 13].

Apart from Rome, the strongest evidence, as always, comes from Pompeii, where it is possible to trace nearly all the circuit of the walls, even though some sections were built over between the earthquake in AD 62 and the volcanic eruption in AD 79. This reveals seven city gates, including the Porta Ercolano (three portals), Porta Vesuvio (two portals), Porta Nola (one portal), Porta Sarno (one portal), Porta Noceria (one portal), Porta di Stabia, (one portal), and Porta Marina (two portals) [38,39]. Although it has been suggested that there was another city gate, the Porta Capua, in the unexplored section of the site to the north-west, excavations have now confirmed that this gate did not, in fact, exist [31, 39]. Under normal circumstances, both the Porta Ercolano and Porta Vesuvio seem to have carried the most traffic to the north, while the Porta di Stabia seems to have carried most of the traffic to the south, at least based on the locations of bars and inns [40]. This situation seems to have been disrupted, however, by the earthquake of AD 62, which led to the collapse of the Porta Vesuvio. This has been included in the dataset, since we would expect it to have been reconstructed, but the effect of adding or removing it is noted below.

Although the quality and quantity of evidence differs from site to site, I have derived estimates for the total numbers and widths of city gates at a number of other sites, which are summarized below.

### The ORBIS model

**Fig 3:**
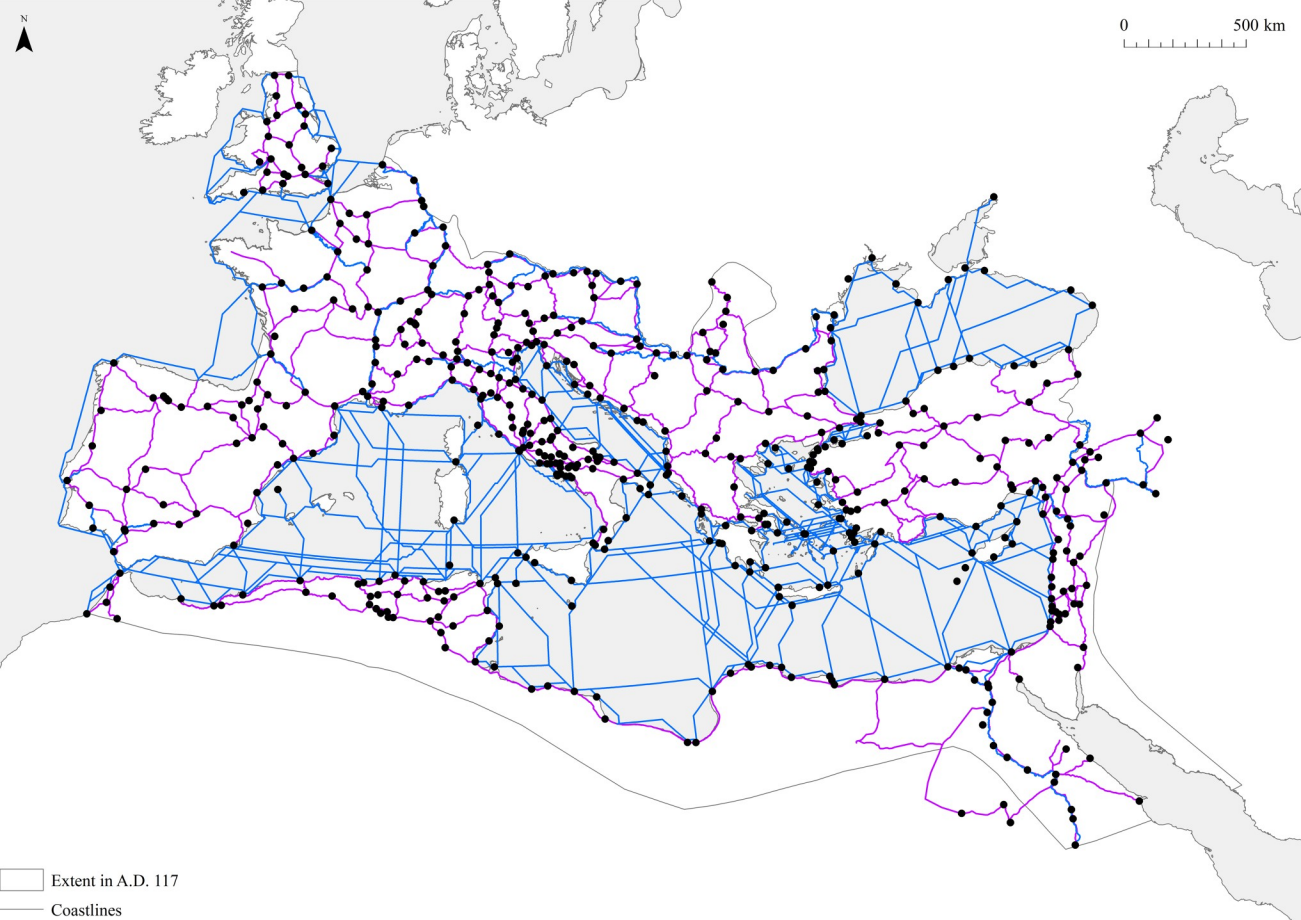
The ORBIS model, which offers a simplified dataset of cities and sea, river, and road networks in the ancient world that roughly reflects conditions around AD 200. The black circles represent cities, while the blue lines represent the sea and river network and the purple lines the road network. The data are derived from https://purl.stanford.edu/mn425tz9757 ((accessed 18th January 2019). No changes have been made to the underlying data.

The network dataset used here is the ORBIS model, which offers a simplified dataset of cities and sea, river, and road networks in the ancient world that roughly reflects conditions around AD 200 (Fig 3) [18, 41]. Although this dataset only includes a limited selection of cities, 678 in total, it does allow us to calculate the distance, time duration, and financial expense associated with different types of travel between different locations for most of the sites that we are interested in here. I have therefore begun by cross-referencing these data with the catalogue of cities referred to above [12].

I then used standard techniques to identify the most important nodes in the network. The measures we are concerned with are degree centrality (the number of edges to a node), betweenness centrality (the number of times a node acts as a bridge along the shortest path between two other nodes), and Eigenvector centrality (the likelihood of a node) being connected to other well connected nodes in the network)) [42]. The last of these assigns a score to each node which is based on the idea that a node will be more important if it is linked to other important nodes in the network. This network has been analyzed in Gephi, assuming that all edges are undirected, but using the cost of moving along these edges in terms of distance (in km), time (in days), and expense as their weights, following other studies and ignoring the season [28]. Once this has been done, it gives a series of metrics for the overall connectivity of sites, allowing investigation of the relationship between the estimated populations of sites and these measures, as shown below.

## Results

**Fig 4:**
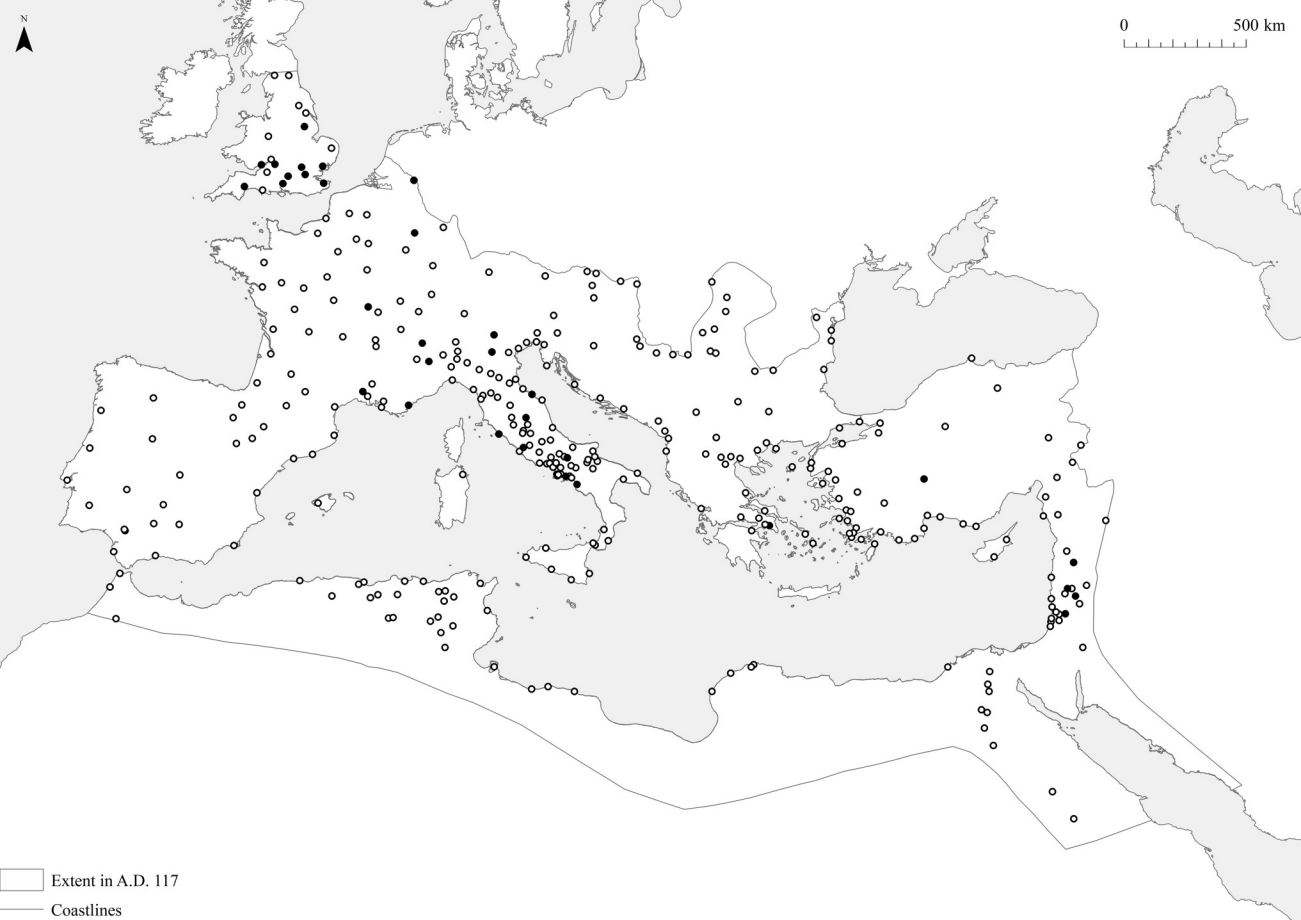
The distribution of the selection of sites discussed in this article. The black circles represent the sites with evidence for the numbers and dimensions of city gates, closed circles the sites that can be located on the ORBIS model.

In total, there is evidence for the numbers and widths of city gates for some 32 sites (this number is simply determined by the amount of evidence available, but it might be expanded in future research). These sites are drawn from throughout the Roman Empire, although they are slightly more concentrated in Italy and the north-west than the rest of the ancient world (Fig 4). This is a relatively small sample of sites, but nevertheless encompasses several orders of magnitude (in terms of estimated population) and includes many of the case-studies discussed in other work [13, 14, 15, 16]. The average number of city gates per city is five, while their average width is just over seven and a half metres.

**Fig 5:**
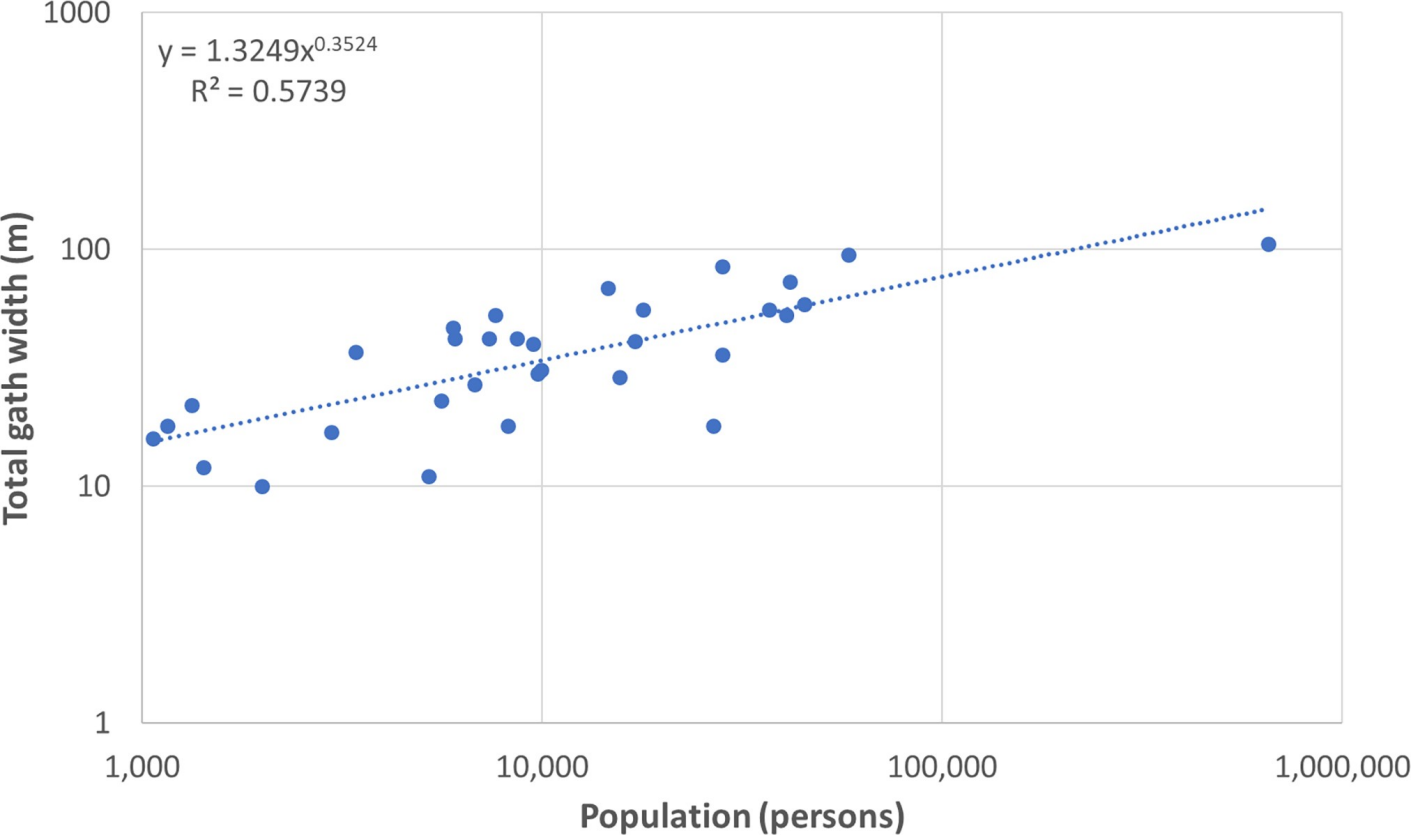
The relationship between the estimated populations and total widths of city gates (in metres) in the selection of cities discussed in this article. Both scales are logarithmic. The total widths of gates increase with population raised to the power of approximately 1/3 (meanwhile both the numbers of gates and the average widths of gates increases with population raised to the power of approximately 1/6).

I then assessed the relationship between the estimated populations of sites and the total numbers and average and total widths of city gates using ordinary least squares regression (Table 2). These results suggest that there is indeed a systematic relationship between the estimated populations of sites and the total widths of their city gates, which is entirely consistent with the view that there are various relationships between the sizes of sites and various aspects of their built environments and socio-economic conditions, including the dimensions of street networks and the magnitude of traffic flows, as discussed above (Fig 5).

**Table 2:** The relationships between the estimated populations and the total widths of city gates, numbers of gates, average widths of gates, and the maximum widths of gates of the sites discussed in this article. All regressions are done using ordinary least squares regressions on log-transformed values.

Dependent variable	Number of cases	Exponent (95% CI)	Pre-factor (95% CI)	R^2^	Significance (P value)
Total widths of gates (m)	32	0.352 (0.244–0.460)	1.324 (0.480–3.656)	0.574	<0.0001
Numbers of gates	32	0.183 (0.087–0.279)	0.935 (0.379–2.307)	0.314	0.0009
Average widths of gates (m)	32	0.168 (0.048–0.288)	1.432 (0.470–4.366)	0.202	0.0098
Maximum widths of gates (m)	32	0.165 (0.063–0.267)	1.726 (0.663–4.493)	0.249	0.0036

Several aspects of this relationship stand out. In the first place, it is worth noting that it is extremely sublinear, meaning that, although the total numbers and widths of city gates does increase with population, it does so only very slowly. This suggests that city gates were used more intensively in larger sites, in line with other studies of infrastructure, so existing city gates would have accommodated some of the augmented traffic [15]. As noted above, although we obviously have to be cautious about making too much of these results, since we expect the total numbers and widths of city gates to be a reflection of the average rate of traffic flow, we might then be able estimate the total volume of traffic by simply multiplying the average figure by the size of the total population. If this is correct, this raises the interesting possibility that the total volume of traffic would have been slightly superlinear, meaning that the numbers of people and total amounts of material passing through them would have increased a little faster than the population of sites, reflecting the social and economic gains that come with agglomeration. This suggests that overall ratios of imported to local wares should also scale with population, controlling for the distance over which the goods had to travel from manufacture to consumption locations. Interestingly, these results are consistent with contemporary evidence for how the numbers of social contacts increases with population in modern cities, based on the numbers of telephone calls, despite the fact that they use very different kinds of data [30].

It is also interesting to observe that both the total numbers and average widths of city gates increase with population at about the same rate, approximately 1/6, suggesting that half of the gains went into the provision of more entrances and exits into cities and half into the construction of wider city gates that could accommodate more traffic. This is in direct contrast to the common assumption that the number of city gates depended on the number of approach roads and not on the numbers of inhabitants or the size of the city [31]. Although the idea that the widths of city gates increased as cities grew larger is somewhat surprising at first, since we would not expect any ancient city to have grown fast enough to have justified the jump from one to two lanes in each direction, this might be explained by the increasing tendency to divide traffic into separate conduits for pedestrians and vehicles, reflecting their different speeds.

The results also indicate that both the average widths of city gates and the average widths of streets within cities increased at around the same rate, with an exponent of 1/6 in both cases, making them consistent with earlier findings [15]. This is especially remarkable, since there is only a partial overlap between the two datasets (only 20 sites are in common). This implies that both interaction rates within cities and between cities and the surrounding regions scale similarly with population, allowing us to integrate existing theories about the internal and external dynamics of cities for the first time.

Although the R^2^ of the overall relationship is high for historical and archaeological data, it nonetheless reflects a relatively high degree of variation. As we have argued elsewhere, the residuals result from a wide range of factors, including both error in how the numbers of residents have been estimated (which itself includes errors in how the sizes and densities of sites have been measured or estimated) and in how the numbers and widths of city gates have been measured [15]. However, it might be possible to use these residuals as an expression of the overall standing of sites, after taking their size into account. An interesting example of this is that, although including or excluding the Porta Vesuvio does alter the residual for Pompeii, this effect is marginal (remaining around -0.05). This suggests that the site might have been only slightly more congested, in terms of transport infrastructure such as city gates, than other cities of the same size. One wonders what implications this might have had for evacuation during the volcanic eruption.

The pre-factor of the overall relationship is 1.32 m. Allowing for some error, this conforms well with ruts from Pompeii, which indicate that the largest carts to use the streets with any regularity were between 1.32 and 1.47 metres, potentially reflecting a situation in which only one vehicle was able to pass through at a time [32]. This also fits well with earlier work on the street networks of a selection of cities, which suggests a baseline street width of a little more than two meters, consistent with the width of a single lane road designed to accommodate a fixed-axle wagon [15, 32]. This supports the idea that ancient cities were quite congested, compared to modern experience [15].

**Fig 6:**
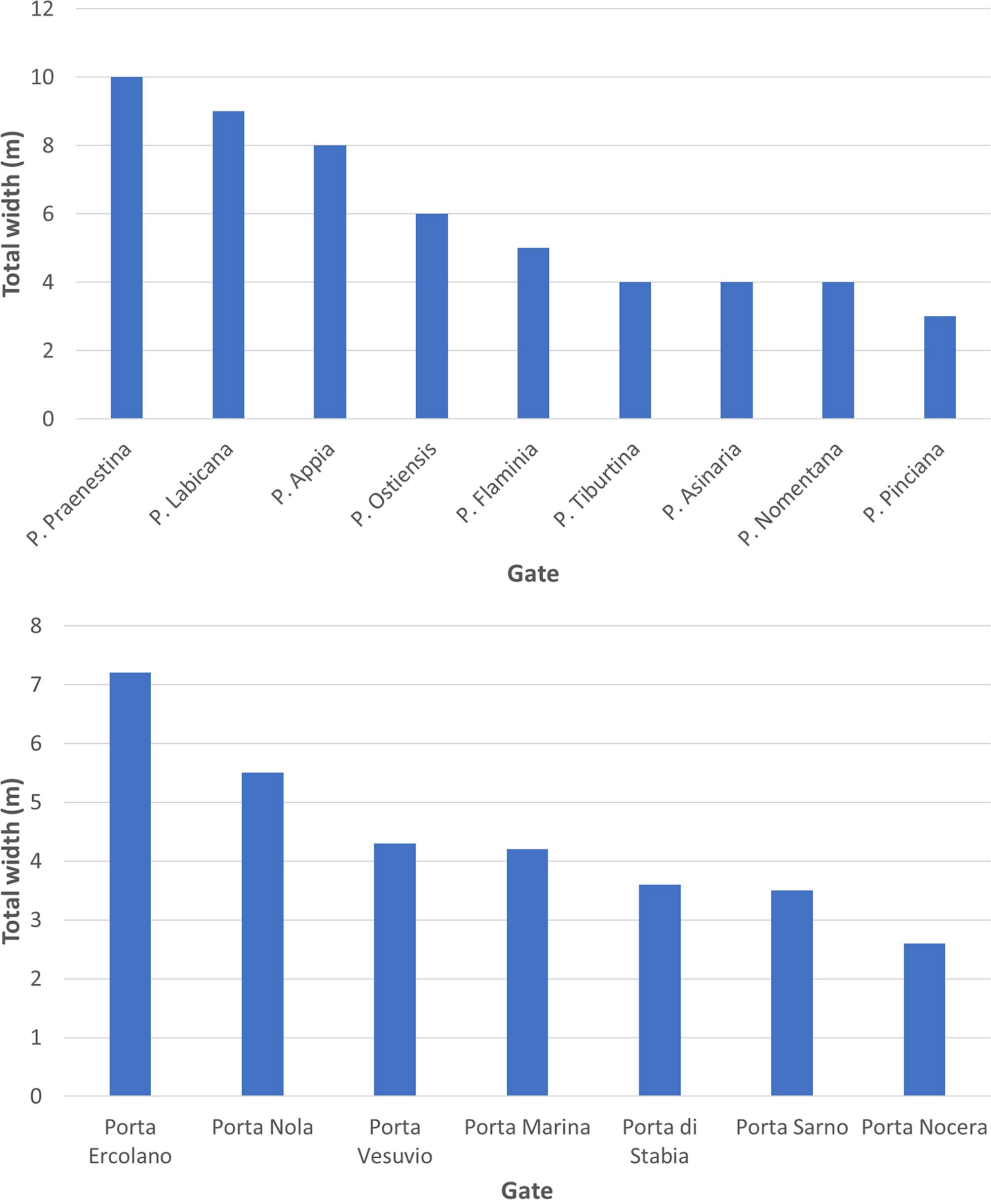
The distribution of the widths of city gates in two cities. Above: Rome; below: Pompeii.

Although we do not have evidence for all cities, there are two cases, Rome and Pompeii, that allow us to investigate the distribution of traffic across city gates within the settlement. These both seem to be close to a power-law, potentially reflecting the fractal nature of traffic flows in modern cities (Fig 6).

It is possible to estimate the network values of 341 sites, which are distributed throughout the ancient world (this number simply being a product of the number of sites included in the ORBIS model; this includes all but one of the sites with city gates mentioned above). The results are somewhat disappointing, in that there is no evidence for a strong relationship between the estimated populations of sites or any of the centrality measures referred to above (Table 3). This is probably caused by a combination of the fact that the ORBIS model only includes a fraction of the actual sea, river, and road networks that must have once existed, and the fact that we are only able to derive rough estimates of the cost of moving across these networks. This means that, while the network is sufficiently detailed to estimate general travel times or to model the spread of various phenomena, it is not detailed enough to explore the complex interactions between the sizes of sites and individual traffic flows.

**Table 3:** The relationship between the estimated populations and the degree centrality, betweenness centrality, and Eigenvector centrality of the sites discussed in this article. All regressions are done using ordinary least squares regressions on log-transformed variables. The low correlations are likely the result of the incomplete data available for ancient sea, river, and road networks.

Dependent variable	Number of cases	Exponent (95% CI)	Pre-factor (95% CI)	R^2^	Significance (P value)
Degree centrality	341	0.105 (0.070–0.140)	1.202 (0.873–1.656)	0.090	<0.0001
Betweenness centrality	341	0.288 (0.135–0.441)	84.918 (21.439–336.357)	0.041	<0.0001
Eigenvector centrality	341	0.180 (0.113–0.247)	0.008 (0.004–0.015)	0.078	<0.0001

## Discussion

At the most fundamental level, city gates can be taken as a measure of interaction rates, since we would expect them to reflect the volume of people, goods, and information flowing into and out of cities. As a result, when taken together, the results of the present study could be interpreted as a reflection of the extent to which settlements and their hinterlands interacted, the scale of surpluses that could be exchanged between them (the largest of which would be water, food, and fuel), and the extent to which they can be regarded as being energetically interdependent. Although this view of ancient cities is very far from the classic accounts of ancient cities, which saw them as cellular, self-sufficient communities, it is more in line with recent ideas [43, 44, 45,46]. As Dermody et al. have argued, the growth of urbanism might have led many cities to overshoot their ecological carrying capacities, so that they depended on their ability to offset deficits in one region with surpluses in another, essentially linking heterogeneous environments through trade [12, 46]. This is also consonant with recent studies of ancient markets, which suggest that, while the ancient world was not as integrated as modern markets, it nonetheless operated as a comprehensive Mediterranean market [45].

This has important implications for our view of the economic profiles of sites, including the extent of their specialization and diversification, since the data suggest that sites could have experienced a sufficiently large increase in traffic with increases in size to allow them to establish different local and regional roles. This raises the question of whether we can use the residuals to give us an index of how much traffic came into or out of each city, above and beyond what we would expect for a city of its size, and therefore as a reflection of its overall specialization and diversification, especially in terms of manufacturing, services, and trade. This is something that needs to be investigated in future work.

The results of the present study might also have a bearing on our overall view of commuting, migration, and social networks. The traditional assumption has been that, since we would expect most people to have lived where they worked, there would have been no commuter traffic. Meanwhile, although it is normally assumed that urban growth relied on large amounts of migration (because of the likely mismatch between birth and death rates in cities), reasonably little is known about this before the modern era. The results discussed above, however, suggest that interaction rates not only increased with size, but also did so at a consistent rate, supporting the idea that flows of people were more common than usually thought. If this is true, it has implications for our understanding of the social networks of ancient settlements, since it implies that they had relatively heterogeneous populations.

Finally, the results might shed some light on the mechanisms through which ancient cities expanded or contracted, since they suggest a systematic relationship between the estimated populations of sites and their potential traffic flows, which is more in keeping with preferential attachment than random growth. This is consistent with the rank-size distribution of cities in the Roman world, which is approximately log-normal (with a value of less than 0.1 for Drennan and Peterson’s test of convexity and concavity), suggesting that these cities functioned as single entity [47]. One of the novel aspects of the new data, however, is that they indicate that this process is not just a result of modern conditions, such as capitalism and industrialization, and might be a general feature of both ancient and modern cities.

## Conclusions

This article has asked whether we can use the built environment to estimate traffic flows, concentrating on transport infrastructure such as city gates. I have presented results that suggest that there is a systematic relationship between the estimated populations of sites and both the total numbers and average widths of city gates, which might be taken as a proxy for traffic flows. This relationship is extremely sublinear, indicating that city gates were used more intensively as cities increased in size, in line with wider theoretical and empirical work. However, since we would expect these city gates to be a reflection of the average rate of traffic flow, we would also expect the total volume of traffic to have been slightly superlinear, reflecting the social and economic gains that come with agglomeration. Another striking aspect of the results is that the average widths of city gates and the average widths of streets increased at the same rate, suggesting that the interaction rates between individuals in cities, and between cities and their surroundings, scale similarly, allowing us to integrate existing theories about the internal and external dynamics of cities for the first time. In particular, the article extends recent theoretical and empirical work on ancient and modern cities by showing that outward-facing transport infrastructure functions in a similar way to the internal street networks documented in earlier papers. This raises the questions of how unique ancient cities were in this respect and whether we can take these relationships as a universal feature of both ancient and modern settlements. This article has also highlighted the importance of investigating the nature and scale of the connections between ancient cites, as well as the need for a much more comprehensive model for the transport network of the ancient world and more detailed evidence for the movement of individuals and commodities through human remains and small finds (although such an attempt will be a considerable undertaking). In the meantime, this article provides a simple approach that can be used to guide future work. It can also be applied to any other contexts where cities were surrounded by walls or other defences, e.g. medieval Europe, imperial China, etc. [48]. This offers a new way of exploring the connectivity and integration of ancient cities, contributing to general theories about how settlements operate across space and time.

## Supporting information

S1 Appendix The numbers, average widths (m), maximum widths (m), and estimated total widths (m) of city gates in selected cities in the Roman Empire (Sheet 1) and the degree centrality, betweenness centrality, and Eigenvector centrality of additional cities in the same region and period (Sheet 2). (XLSX)
